# Inequalities in the progress of multiple chronic conditions: A systematic review of longitudinal studies

**DOI:** 10.1371/journal.pone.0263357

**Published:** 2022-02-03

**Authors:** Rolla Mira, Tim Newton, Wael Sabbah

**Affiliations:** Faculty of Dentistry, Oral & Craniofacial Sciences, King’s College London, London, United Kingdom; Chiang Mai University, THAILAND

## Abstract

The objective of this review is to assess the impact of socioeconomic factors on the progress of multiple chronic health conditions (MCC) in Adults. Two independent investigators searched three databases (MEDLINE, EMBASE and LILACS) up to August 2021 to identify longitudinal studies on inequalities in progress of MCC. Grey literature was searched using Open Grey and Google Scholar. Inclusion criteria were retrospective and prospective longitudinal studies; adult population; assessed socioeconomic inequalities in progress of MCC. Quality of included studies and risk of bias were assessed using the Newcastle Ottawa Quality Assessment Scale for longitudinal studies. Nine longitudinal studies reporting socioeconomic inequalities in progress of MCC were included. Two of the studies had poor quality. Studies varied in terms of follow-up time, sample size, included chronic conditions and socioeconomic indicators. Due to high heterogeneity meta-analysis was not possible. The studies showed positive association between lower education (five studies), lower income and wealth (two studies), area deprivation (one study), lower job categories (two studies) and belonging to ethnic minority (two study) and progress of MCC. The review demonstrated socioeconomic inequality in progress of multiple chronic conditions.

**trial registratiom**: The review protocol was registered in the International Prospective Register of Systematic Reviews (CRD42021229564).

## Introduction

Multiple chronic conditions (MCC) have been recognised as a major global public health concern with a continuously increasing prevalence especially among older adult [1]. The increased prevalence of MCC became a burden on health sectors due to adverse health outcomes leading to higher rates of hospitalisation and use of healthcare [2].

Multiple chronic conditions are defined as the presence of two or more chronic diseases. While hereditary factors and early life contribute to their co-occurrence, there is also considerable role for behavioural and psychological factors, all are socially patterned [3–5]. The most common behavioural factors that contribute to multiple chronic conditions are smoking, poor nutrition, lack of physical activity, obesity, and alcohol consumption [6,7]. Furthermore, socioeconomic factors were found to impact chronic conditions and related behaviours through different pathways [8].

The distribution of multiple chronic diseases varies from one population to the other depending on the type of studies used in assessing them and the data sources [1]. Multiple chronic conditions could include a broad range of diseases, most commonly, diabetes, hypertension, coronary heart disease, chronic kidney disease, chronic pulmonary disease, thyroid disease, heart failure, obesity, stroke, cancer, dementia, depression, metal health problem and lower back pain [7,9–11].

Unsurprisingly, there is a surge in studies examining the determinants of multiple chronic conditions and their progression to enable tackling this public health problem [12]. Earlier reviews, mostly based on cross-sectional studies, reported socioeconomic inequalities in MCC based on household income, area deprivation, education, and socioeconomic class [7,13–15]. Only one review was limited to longitudinal studies based on primary care data, but inequality was not the focus of the review with only one paper reporting education inequality in MCC [3]. In the USA, data from the Health and Retirement Study demonstrated ethnic inequalities in the progress of MCC with non-Hispanic Blacks having higher rates than other ethnic groups [16]. Additionally, several longitudinal studies examined the potential risk factors for multiple chronic conditions. These included sociodemographic factors (age, gender, ethnicity, education, income) among others [9,11,17]. Furthermore, several behavioural factors contribute to the occurrence and progression of MCC, these include physical activities, diet, smoking, alcohol consumption and body mass index (BMI) [11,17–19]. While the role of socioeconomic factors as the underpinning determinants of the prevalence of MCC [8,20] is well-established, there is less research exploring the determinants of the progress of MCC. Therefore, the present review sought to determine the impact of socioeconomic factors on the progress of Multiple Chronic Conditions in Adults.

## Methods

This systematic review was conducted in adherence with the guidelines of Preferred Reporting Items for Systematic Reviews and Meta‐analyses (PRISMA) statement [21]. The review protocol has been registered in the International Prospective Register of Systematic Reviews (PROSPERO) (Registration number CRD42021229564).

### Eligibility criteria

PRISMA 2020 guidance was used as criteria for eligibility which include PECO, as ‘P’, participants ‘adults’ (+18), ‘E’, exposures ‘socioeconomic factors’, ‘C’, comparison groups and ‘O’, outcomes ‘progress of MCC’.

### Inclusion criteria

- Retrospective and prospective longitudinal studies.
- Participants were only adult population (18 years old and above).
- Exposure included any indicator of socioeconomic factors at least one time in the duration of the study, for example (household income, wealth, unemployment, education, early life socioeconomic factors, social status, deprivation).
- The outcome is progress of multiple chronic conditions from 1 or 2 at baseline to multiple conditions at follow-up stages.

### Exclusion criteria

- Randomized Controlled Trails
- Controlled Trails without Randomization
- Interventions with before and after comparison.
- Cross-sectional studies.
- Case-control studies.
- Studies which included participants under 18 years old.
- When no indicator of socioeconomic factors has been reported in the study.

### Information sources

**Fig 1:**
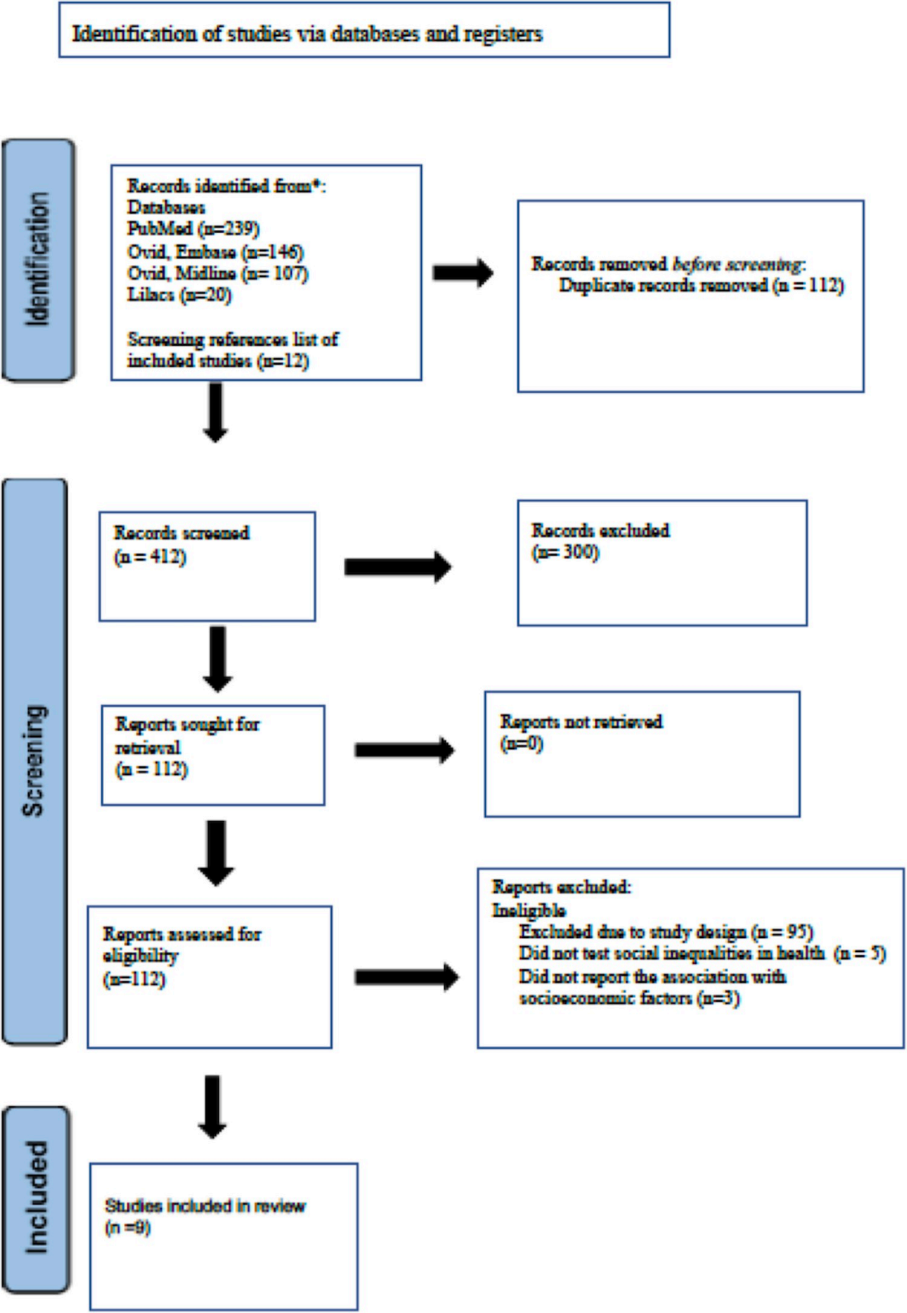
Flow diagram of selected studies.

Two independent reviewers conducted the literature search using three databases (PubMed, Cochrane library and Ovid) up to August 2021. Published and accessible papers were considered in the literature review. Authors were also contacted for grey literature. Papers were filtered by their title and abstracts for relevance. Finally, papers were included by reading the entire articles (Fig 1). All references were obtained in software Endnote X9.

### Search strategy

We used the following search terms to search all studies registers and databases, multimorbidity OR multi-morbidity OR Complex multimorbidity OR Complex multi-morbidity OR multiple chronic conditions OR multi-morbid AND Socioeconomic factors OR Social determinants OR socioeconomic position OR socioeconomic status OR social class OR Education OR wealth OR income OR household wealth OR employment OR poverty OR deprivation AND longitudinal OR prospective studies. Date restrictions were for papers published between 2000 to August 2022. English language restriction was applied.

### Selection process

Eligibility assessment was performed independently in an unblinded standardized manner by 2 reviewers. PRISMA flow diagram was followed to create a flowchart illustrating number of the studies at each stage of the review and reasons for exclusion after assessing the eligibility (Fig 1). Disagreements between reviewers were resolved by discussion to reach a consensus.

### Data collection process

Two reviewers extracted data pertaining to study design, including authors, year of publication, country, and participants’ characteristics (sample size, age, follow-up duration), exposure [socioeconomic indicators], outcomes, results, and conclusions from the included papers.

### Study risk of bias assessment

Risk of bias in the included studies was assessed by two independent reviewers using the Newcastle‐Ottawa Scale (NOS) for longitudinal studies [22]. The Newcastle-Ottawa is a statistical tool used for assessing the quality of studies included in systematic reviews. Each study is judged on eight items, categorized into three groups: 1) selection of the study groups (representativeness of exposed, selection of non-exposed, ascertainment of exposure, outcome not present at start); 2) comparability of the groups (control for confounders); and 3) ascertainment of either the exposure or outcome of interest (assessment of outcomes, follow-up length, adequacy of follow-up). Quality levels are either good, fair or poor. These levels are classified according to a specific score which ranges from zero to nine stars for each article, in which a greater number of stars indicates a higher‐quality study. **Good quality:** 3 or 4 stars in selection domain AND 1 or 2 stars in comparability domain AND 2 or 3 stars in outcome/exposure domain. **Fair quality:** 2 stars in selection domain AND 1 or 2 stars in comparability domain AND 2 or 3 stars in outcome/exposure domain. **Poor quality:** 0 or 1 star in selection domain OR 0 stars in comparability domain OR 0 or 1 stars in outcome/exposure domain.

### Effect measures

Effect measures for the exposures, namely, education, income and wealth, area deprivation, occupation and ethnicity were indicated by odds ratio, hazard ratios, incidence rate ratio, interquartile range.

### Synthesis methods

Data on exposures (education, income and wealth, area deprivation, occupation and ethnicity) and outcomes (progress of multiple chronic conditions over the follow-up period) and effect estimate were collected from the different studies. Data on co-variates adjusted for in each study were also collected. Indicators of socioeconomic factors were included along with follow-up time, sample size, demographic characteristics. Given the high heterogeneity of the included studies, particularly in relation to variations in outcomes, socioeconomic exposures, sample size and follow up periods, it was not possible to conduct meta-analysis of the included studies.

### Certainty assessment

All the studies reported confidence intervals.

## Results

### Study selection

A summary of the results of the study selection is shown in Fig 1. The preliminary electronic search yielded 512 references from three different databases (PubMed, Lilacs, Ovid Midline and Ovid Embase). Searching bibliography of the identified papers yielded 12 studies. After removal of duplicates, 412 references were retained for screening of titles and abstract, then 300 articles were excluded as they were irrelevant. After evaluation of the full reports of the remaining 112 references, only twelve met the inclusion criteria. An additional three studies did not report the association between socioeconomic indicators and progress of MCC [23–25]. The authors of one of these papers [25] reported that there were negative association between education and progress of multiple chronic conditions, but it has not been reported as it will be reported in a subsequent paper. Authors of the other two papers did not respond to our question [23,24]. Therefore, these 3 studies were excluded from the review and nine studies were included. A flowchart illustrating the number of the studies at each stage of the review and the reasons for exclusion after assessing the eligibility is presented in Fig 1. The methodological assessment of the included studies using NOS criteria is presented in Table 1.

**Table 1:** Methodological assessment of included studies using the Newcastle-Ottawa Scales (NOS) for longitudinal studies.

Study (First Author)	Study design	Selection				Comparability	Outcome			Overall score And quality
		Representativeness of the sample	Selection of the non-exposed cohort	Ascertainment of exposure	Demonstration that outcome of interest was not present at start of study	Based on design and analysis	Assessment of the outcome	Was follow-up long enough for outcomes to occur	Adequacy of follow up	
Dugravot, Fayosse [26]	Longitudinal	*	*	*	*	**	*	*		8 Good Quality
Quiñones, Botoseneanu [27]	Longitudinal	*	*	*	*	**		*	*	8 Good Quality
Hussin, Shahar [28]	Longitudinal	*	*	*	*	**				6 poor Quality
Singh-Manoux, Fayosse [29]	Longitudinal	*	*	*	*	**	*	*	*	9 Good Quality
Alaeddini, Jaramillo [30]	Longitudinal	*	*	*	*	**	*	*	*	9 Good Quality
Katikireddi, Skivington [17]	Longitudinal	*	*	*	*	**		*		7 Poor Quality
Melis, Marengoni [31]	Longitudinal	*	*	*	*	**	*		*	8 Good Quality
Quiñones, Liang [16]	Longitudinal	*	*	*	*	**		*	*	8 Good Quality
van den Akker, Buntinx [32]	Longitudinal	*	*	*	*	**	*		*	8 Good Quality

### Study characteristics

The characteristics of the included studies are presented in Table 2. All the included studies were conducted among older adults with the exception of three studies that included adults aged 18 years and over [17,30,32]. The follow-up periods ranged from 11 to 24 years except for three studies with follow-up periods one and half year, two years, and three years, respectively [28,31,32].

**Table 2:** Characteristic of longitudinal studies on socioeconomic inequality in progress of multiple chronic conditions.

Study	Study Design	Country	Population and setting	Age	Exposure	Outcome
Dugravot, Fayosse [26]	Longitudinal study (24 years follow up)	United Kingdom	10,308 at baseline. 6,425 at follow-up	35–55 years old	Socioeconomic inequalities (Education, occupation, literacy and) including three levels: high, medium, low. Models adjusted for (age, race, marital status, and birth cohort	Adverse health outcomes (Multimorbidity, Frailty and Disability) and mortality. Multimorbidity measured by the incidence of two or more of diabetes, coronary heart disease, stroke, chronic obstructive pulmonary disease, depression, arthritis, cancer, dementia, and Parkinson’s disease).
Quiñones, Botoseneanu [27]	Longitudinal study (16 follow up)	United states	10,126 at baseline 8,872 at follow-up	51–55 years old	Ethnicity (non-Hispanic White, non-Hispanic Black, and Hispanic) Sociodemographic factors (gender, education, and BMI)	The evaluation of how multimorbidity develops and progresses over time among middle-aged Multimorbidity was defined as having two or more of seven somatic chronic diseases: arthritis, cancer, heart disease (myocardial infarction, coronary heart disease, angina, congestive heart failure, or other heart problems), diabetes, hypertension, lung disease, and stroke), other heart problems), diabetes, hypertension, lung disease, and stroke)
Hussin, Shahar [28]	Community-based longitudinal study (follow-up 1and a half year)	Malaysia	2,322 at baseline 729 at follow-up	60 years and older	Multi-ethnic Malaysian groups. Sociodemographic data (gender, age, and education)	Incidence and predictors of multimorbidity and stratified participants at baseline or the presence of one chronic disease through list contained 15 chronic diseases.
Singh-Manoux, Fayosse [29]	Longitudinal study (23.7 years follow up)	United Kingdom	10,308 participants at baseline. 8,270 at follow-up	35–74 years old	The role of clinical characteristics (hypertension, hypercholesterolemia, overweight/ obesity, family history of cardiometabolic disease), socioeconomic position (occupational position which is grade of employment as a comprehensive measure that reflects education, occupational status, and income), and behavioural factors (smoking, alcohol consumption, diet, physical activity) All analysis were repeated, replacing socioeconomic position with occupational position then with educational level by measuring the highest qualification on leaving full-time education (no academic qualifications, lower secondary school, higher secondary school, university, higher degree).	Development of cardiometabolic disease (diabetes, coronary heart disease, stroke), cardiometabolic. Multimorbidity (2 or more of cardiometabolic disease), and mortality
Alaeddini, Jaramillo [30]	Retrospective longitudinal study (2002–2015) = 13 years	United states	608,503 at baseline 601,805 at follow-up	>18 years	Diverse population of patients (Iraq and Afghanistan war Veterans) Sociodemographic data (age, gender, race/ethnicity, marriage status (married or not), education, and age.	Investigate the risk factors associated with the emergence and progression of MCCs and predicting MCC transitions at both individual and population levels.
Katikireddi, Skivington [17]	Longitudinal study 20 years follow up	United Kingdom, West of Scotland	4510 at baseline followed up 2604 at follow-up	15–55 years	Five different risk factors (smoking, alcohol consumption, diet, body mass index (BMI), physical activity). Socioeconomic status: Household income Area based deprivation	Development of multimorbidity (2+ health conditions)
Melis, Marengoni [31]	Longitudinal study (follow up 3 years)	Sweden	418 at baseline 390 at follow-up	75 years and over	Social demographic measures: age, gender, living situation, living arrangement, and education). Lifestyle indicators (smoking, alcohol consumption, physical activity), Medical conditions and biomarkers	Estimate the incidence of multimorbidity and identify the possible predictors for multimorbidity. Multimorbidity (Incident cases were defined as subjects with no or only one chronic disorder at baseline who developed at least another chronic disease during the 3-year follow-up.)
Quiñones, Liang [16]	Longitudinal study (follow up 11 years)	United states	17,517 at baseline 15,576 at follow-up	51 years and over	White, Black, and Mexican Americans ethnicities. socioeconomic factors, marital status, and behaviours: household income, physician visits, BMI, self-rated ill health, and education	Progress of multiple chronic conditions. Multimorbidity measured by composite of seven diseases: hypertension, heart disease, diabetes, cancer, lung disease, arthritis, and stroke
van den Akker, Buntinx [32]	Longitudinal study (2 years follow up)	Netherland	3745 at baseline 3551 at follow-up	20 years and older	Sociodemographic factors (age, gender, education). Health insurance (private or public) Number of chronic conditions at the start of the follow up	Assessment of risk for developing multiple chronic conditions over a short follow-up period.

All selected studies defined multimorbidity (multiple chronic conditions) as the presence of 2+ chronic conditions. However, there were variations in the indicators of socioeconomic inequalities such as education, household income and wealth, occupation and ethnicity. It is worth noting that some studies focused on other determinants of the progress of MCC, for example individual’s lifestyle style factors (smoking, physical activity, alcohol consumption, diet, and body mass index). Summary of the association between socioeconomic factors and progress of multiple chronic conditions are presented in Table 3.

**Table 3:** Association between socioeconomic factors and progress of multiple chronic conditions.

Study	Independent predictor	Predictor	Description of the predictor	Adjusted measure of association (95%CI)	Covariates	Results	Comments
Dugravot, Fayosse [26]	Multiple chronic conditions (MCC)	Education Occupation literacy	Low Medium High (compare low to high)	Hazard ratio (HR) for transition from healthy status to MCC: education: 1.24 (1.13, 1.35) Occupation: 1.54 (1.37, 1.73) Literacy: 1.11 (1.07, 1.14)	Age, gender, ethnicity, and marital status at 50 years old	Lower socioeconomic status was significantly associated with higher hazard ratio for transition from healthy status to MCC.	Participants were only assessed at age 50 years old (only at baseline). 1694 participants developed MCC.
Quiñones, Botoseneanu [27]	MCC	Hispanic Black, non-Hispanic White, and Hispanic Americans Socioeconomic factors (education)	Ethnicities (Hispanic Black, non-Hispanic White, and Hispanic Americans) Education (number of school years completed	Incidence Rate Ratio (IRR): Education: 0.95 (0.93, 0.95)	Gender and Body-mass index (BMI)	For each additional year of education, the rate of accumulation of chronic condition decreases by 0.9 unite in other words, greater educational attainment is significantly associated with slower accumulations of chronic disease. The more BMI weight increase, the accumulation of chronic conditions increases by 1.011	The results were limited to certain chronic conditions
Hussin, Shahar [28]	MCC	Multi-ethnic Malaysian groups and education	Education (years of schooling) No schooling 1–6 7–11 12years and above	Association between education and multiple chronic conditions was insignificant, OR 1.29 (0.55, 3.02)	Age, gender, smoking, cognitive function, lifestyle, and chronic condition at baseline.	No socioeconomic inequalities. Females, smokers, and individuals with inadequacy in preparing food were more likely to develop multimorbidity than their respective counterparts.	Follow up period was not enough to establish more accurate results
Singh-Manoux, Fayosse [29]	MCC	Occupational position and educational level Behavioural factors Clinical profile	Occupational position and educational level: high versus low. Clinical profile: Scale from 0–4 0 = highest/heathiest 4 = lowest/ unhealthiest	HR of lowest occupational position to progress from no disease to one disease 1.42 (1.23, 1.64), and one disease to multiple conditions 1.54 (1.10, 2.15). HR for lower education to move from healthy to one disease 1.52 (1.30, 1.77) And from one disease to multiple conditions 1.48 (1.04, 2.10)	Age, sex, race (White, non-White), marital status (single, non-single), and birth cohort (4 categories: 1935, 1936±1940, 1941±1945, >1945) at age 50	The lower levels of socioeconomic factors were significantly associated and showed higher hazard ratio to develop MCC. The lowest level of behavioural and clinical factors was significantly associated with multimorbidity.	Risk factors were only assessed at age 50 years old and changes in any risk factors due to treatment or life modification was not assessed.
Alaeddini, Jaramillo [30]	MCC	Diverse population of patients (Iraq and Afghanistan war Veterans) Sociodemographic data race/ethnicity and education	Race/ethnicity (white, black, Hispanic, Asian, and Native American), education (education at the time of military discharge or last deployment was classified as less than high school, high school, some college, college, and post baccalaureate)	Significance Level was set at 0.01 in the paper. There was no significant association with education, ethnicity. When we reduced significance level to P < 0.05, only being married was significantly associated with MCC	Age, gender, race/ethnicity, poverty status, date and type of care received (e.g., primary care, specialty care), and ICD-9-CM diagnostic codes to identify conditions for which care was received	No association were found between sociodemographic factors and MCC except for marital status.	Limited to four chronic conditions only (depression, Posttraumatic stress disorder, Hypertension, and Low back Pain.
Katikireddi, Skivington [17]	MCC	Socioeconomic status: Household income Area deprivation five different risk factors (smoking, alcohol consumption, diet, body mass index (BMI), physical activity)	Area deprivation: Least, intermediate and, most Smoking: Never, Current, Ex) Alcohol consumption: No excess, excessed, none/ex Diet: Every day, some days, never BMI: Healthy Overweight Obese Morbidly obese Underweight Physical activity: 3days 1-3days none	Area deprivation: least deprived had OR 1.46 (1.26, 1.68) Lower income OR 1.53 (1.25, 1.87) Smoking: 1.57 (1.37, 1.80) Alcohol consumption: 1.49 (1.26, 1.76) Diet: 1.45 (1.24, 1.71) BMI: 1.98 (1.50, 2.62)	Age Gender cohort, previous MCC, time between waves, and cohort*gender interaction)	The socioeconomic disadvantages are positively associated with the development of MCC as people who lives in the most deprived areas are 1.46 more likely to develop MCC than others	The measurement of diet was only limited to vegetable and fruit consumption which gives inaccurate results about MCC, other dietary items may be more related to MCC such as salt and fat saturated food
Melis, Marengoni [31]	MCC	Education Lifestyle Medical condition Biomarker	Education was measured by the maximum years of formal schooling, and this variable was dichotomized (Less or equal 8 years or more than 8 years). No chronic condition at baseline. One chronic condition at baseline.	Education was not associated with progress of MCC. Adjusted OR of age 1.09 (1.01, 1.17) Adjusted OR of Worse cognitive abilities 1.22 (1.00, 1.48)	Sociodemographic data: age, gender, living situation and living arrangement. Lifestyle: physical activity, smoking, and alcohol drinking.	No association between education and incidence of MCC Age and cognitive abilities were the only significantly associated variables with MCC	There were very few significant associations due to the small sample size and the characteristic of patients were only assessed at baseline.
Quiñones, Liang [16]	MCC	Socioeconomic factors (Household income and education) White, Black, and Mexican Americans ethnicities	Socioeconomic factors (Household income reported per 1,000s of dollars. Education was measured as a continuous variable denoting year of schooling (range 0–17). Ethnicities (White, Black, and Mexican Americans)	Higher education and income were negatively associated with the progress of MCC	Age, gender, ethnicity, marital status, physician visits and BMI	Income and education inequality. Black individuals reported highest rate of developing MCC.	The study accounted for time variant factors including income. Chane in income over time was associated with MCC.
van den Akker, Buntinx [32]	MCC	Education, Health insurance (private or public) Number of chronic conditions at the start of the follow up	Education: low level secondary high level Number of diseases at the start of the follow up none one two or more	Highest level of education showed OR 0.42 (0.54, 0.95) for developing MCC. Having two or more chronic conditions at the start of the follow up was positively associated with the progress of MCC OR 1.98 (1.58, 2.48)	Sociodemographic factors (age, gender, education). Health insurance (private or public) Number of chronic conditions at the start of the follow up	Occurrences of multimorbidity increased with old age, lower level of education, public insurance and having 2 or more conditions at the start of the follow up.	Higher education was negatively associated with the progress of MCC and having chronic conditions at the start of the follow up was positively associated with the progress of MCC

### Risk of Bias in the included studies

Based on the criteria of Newcastle-Ottawa scale (Table 1), two out of the nine studies had score of 9, five had score 8, one scored 7 and one scored 6. Seven papers were rated as good quality and two as poor quality as they did not score high enough in the outcome domain [28,31]. A good quality study requires 3 or 4 stars in selection domain and 1 or 2 stars in comparability domain and 2 or 3 stars in outcome domain. While poor quality requires 0 or 1 star in selection domain or 0 stars in comparability domain or 0 or 1 stars in outcome/exposure domain. Difference in the rating between the studies was mainly due to different scores in the outcome domain. All the studies selected the non-exposed cohorts from the same community of the exposed cohorts. Two studies accounted for ethnicity, age, gender, and socioeconomic status while the remaining studies accounted for all these factors but not ethnicity. Three were linked to medical records for the outcome assessment [26,30,32] and two had clinical assessment [29,31]. Three out of the nine studies had short follow-up periods, one year and a half, two years, and three years [28,31,32]. Finally, all the studies did not provide any description of blind assessment of MCC.

## Results of individual studies

Association between the socioeconomic factors and progress of MCC (Table 3).

### Education

Eight studies examined the relation between individuals’ educational level and progress of multiple chronic conditions but reported different results. Significant association were reported in five studies, participants with low education had 1.24 hazard ratios for developing MCC [26], while in another study the hazard was 1.54 for developing the first condition and 1.48 for developing multimorbidity among those with low educational level [29]. In a study in USA, for each additional educational year the rate of chronic conditions accumulation decreased by 0.95 [27]. The same author in another study found that higher education was negatively associated with the progress of MCC (coefficient -0.053) [16]. Similarly, in the Netherland, multimorbidity was lower among people with middle and high education than those with low education with odds ratio 0.82 and 0.42, respectively [32]. Three studies reported no significant relation between education and progress of multiple chronic conditions [28,30,31].

### Wealth and household income

In the USA there was a negative association between higher income/ greater wealth and progress of multiple chronic conditions [16]. In another study in Scotland, persons with lower income had higher odds for progress of MCC (OR: 1.53) [17].

### Area deprivation

One study examined area deprivation among three categories: least, intermediate, and most deprived. These categories were measured by Carstairs scores for postcode sectors which includes four indicators of socioeconomic status (car ownership, male unemployment, overcrowding, and low social class). Persons who lived in the most deprived areas were more likely to develop multiple chronic conditions with the odds for those living the most deprived 1.46 compared to those in least deprived areas [17].

### Occupation

Only two studies used occupation as an indicator of socioeconomic position [26,29]. In both studies being in lowest occupational categories was significantly associated with moving from healthy status to multimorbidity [29] and from one condition to multimorbidity [26].

### Ethnicity

Three studies examined the association between ethnicity and progress of multiple chronic conditions. Two studies based on longitudinal national survey in the USA found that non-Hispanic Blacks had higher rates of multiple chronic conditions at baseline and the end of the study (1.6 and 2.67) [16], and (1.3 and 3.3) [27] than non-Hispanic White and Hispanic/ Mexican Americans. Another study conducted among USA veterans found no significant difference by ethnic groups in multiple chronic conditions [30].

### Results of syntheses

In general, the review findings demonstrated the impact of socioeconomic conditions on the progress of MCC. According to NOS, the included studies were classified as good quality apart from two studies that were classified as poor quality [17,28]. The included studies showed high heterogeneity which prevented meta-analysis conduction.

Only one study explicitly examined the mediating role of behaviours in deprivation inequality in accumilation of MCC over 20 years [17]. After accounting for behaviours, including smoking, diet, alcohol consumption, BMI and physical activities, deprivation inequality was attenuated by 40.8%. Another study argued that ethnic differences in progress of MCC were attenuated by socioeconomic factors [16]. The rest of the included studies examined the role of different factors in the progress of MCC, such as behavioural factors, BMI, cognitive function, insurance and existing chronic conditions at baseline, but none of them examined whether these factors could explain socioeconomic inequalities in the progress of MCC [26–32].

## Discussion

Socioeconomic inequalities in progress of multiple chronic conditions (MCC) were examined in previous reviews but these reviews were limited to either cross-sectional studies [14,15] or longitudinal studies based on primary healthcare with no focus on socioeconomic inequality [3]. This is the first review that included population-based longitudinal studies on socioeconomic inequalities in progress of multiple chronic conditions among adults. The systematic review included nine longitudinal studies, three were conducted in United Kingdom [17,26,29], three in the United States [16,27,30], one in Sweden [31], one in Malaysia [28] and one in the Netherland [32].

There was high heterogeneity in the included papers, particularly in terms of selection of socioeconomic factors. for example, education level, wealth and household income, occupation and ethnicity, and the outcomes used in each study such as, number of chronic conditions, the development of certain chronic conditions. Furthermore, some studies examined progress from healthy status to multimorbidity, or transition from one condition to two or more. In addition, there were also variations in length of follow-up periods, sample size and the population. These variations did not allow pooling of the results from the included papers.

Most of the included papers were judged to have a low risk of bias according to Newcastle-Ottawa Scales apart from two papers that were rated as high risk of bias as they did not meet the criteria in the outcome domain [17,28]. It is worth noting that five studies relied on objective assessment of the outcomes as three used medical records [26,30,32] and two had clinical examination of the outcomes [29,31]. Three papers had high attrition rate [17,26,28], but one was rated good quality [26].

The follow-up periods had a significant role in the development of multiple chronic conditions, as their progress increase over time, the longest follow up period was 24 years reported in two studies conducted in the UK [26,29]. The long follow-up period allowed adequate time to report the incidence of certain multiple chronic conditions such as diabetes, coronary heart disease and stroke. On the other hand, the shortest follow-up period was found in three studies, one and half year [28], two years [32] and 3 years [31]. Undoubtedly, these periods were not long enough to show the progress of multiple chronic conditions. Additionally, two studies had the smallest sample size compared to other studies as they only examined 390 and 729 [28,31], respectively.

Generally, the reviewed studies provided evidence of a longitudinal relationship between education, wealth and income, area deprivation occupation and ethnicity on the one hand, and progress of multiple chronic conditions on the other, a relationship that existed even after accounting for other risk behaviours in most of the studies. Pervious systematic reviews highlighted education, income and deprivation inequalities in MCC, but they were mostly based on cross-sectional studies [14,15] or a combination of both longitudinal and cross-sectional studies [13]. The selection of socioeconomic factors included in this review also varied between different countries. For example, in USA race and ethnicity are always used as indicators of socioeconomic position. Two of the studies in this review reported ethnic inequalities in the progress of MCC [16,27] and argued the ethnic inequalities could be explained by socioeconomic status [16]. On the other hand, in the United Kingdom occupation is always used as it reflects social status in the society, levels of power and control at workplace [20]. Unsurprisingly, two of the included studies that used longitudinal data from UK reported occupational inequalities in progress of MCC [26,29].

All the studies included number of chronic conditions to evaluate their progress among adults over time. However, variations in the number of included chronic conditions, and whether participants were health at baseline, resulted in some inconsistency in assessing their progress.

Certain chronic conditions were repeatedly examined in most of the papers such as, diabetes, coronary heart disease, cancer, stroke, and hypertension [26,29,31]. On the other hand, the included studies used different indicators of socioeconomic position (SEP) such as education, wealth and income, area deprivation, occupation and ethnicity.

Education level was used as indicator of SEP in eight studies. Five studies reported negative association between the progress of multiple chronic conditions and education level, as individuals with low educational degree were at greater risk of MCC compared to those with higher degrees [16,26,27,29,32] On the other hand, three studies found no significant association between education level and incidence of multiple chronic conditions [28,30,31] While education does not necessarily reflect current wealth, material ability, employment status or job classification, particularly among older adults at retirement age, it usually reflects early and mid-life socioeconomic conditions which impact chronic conditions, many of them result from accumulation of risk factors over the span of life [8]. Education also enables between-countries comparison.

Personal wealth and household income were used in two studies [16,17]. One of these studies used household income as inflation reported per 1,000s of dollars [16], while the other study reported household income by number and age of people living in the household [17]. Both studies showed positive association between low household income and progress of multiple chronic conditions. These indicators of wealth and income reflect current socioeconomic position and material ability and are clearly relevant to the progress of MCC over time. Inadequate financial resources at baseline will undoubtedly impact ability to afford and access healthy diet, preventive services and other health promoting resources in the community. On the other hand, financial adversities are linked to anxiety, worries and depression, and could prompt individuals to adopt unhealthy behaviours such as smoking, excessive drinking and unhealthy eating. The psychological impact of financial distress could also affect the body systems and the biological markers of many of the chronic condition [8,20]. The mechanisms will subsequently lead to progress of chronic conditions throughout the follow-up period. This was particularly evident in studies with long follow-up time [16,17].

Only one study used area deprivation as indicator of socioeconomic position [17] and found that people living in the most deprived areas are more likely to have multiple chronic conditions than those living in intermediate or least deprived areas. While area deprivation does not necessarily reflect individual socioeconomic status, it reflects individual’s ability to choose destination of residence, which could be linked to wealth and financial ability [33]. Furthermore, people living in deprived areas often have inadequate access to health promoting environment. It is worth noting that the study by Katikireddi, Skivington [17] explicitly examined the mediating role of health-related behaviours in deprivation inequalities in the progress of MCC [17]. The study reported that inequalities in MCC were reduced by 40% after accounting for behavioural factors. A finding that, to some extent support the aforementioned theory on how area deprivation influences progress of chronic condition.

In the United States, ethnicity is often used as a marker for socioeconomic position. Two studies that used national longitudinal data found that African Americans were more likely to experience progress of MCC than Whites [16,27]. On the other hand, another study did not find relationship between different ethnicities and the progress of MCC among American veterans [30]. It is worth noting that several studies argued that ethnic differences in health could be explained by socioeconomic differences between ethnic groups, early life circumstances, area of residence, and perception of discrimination particularly among ethnic minorities [34,35]. Indeed, in one of the studies included in this review Quiñones, Liang [16] argued that income and education explained part of the ethnic inequalities in the progress of MCC among American adults. Aside from these two studies [16,17], none of the included studies examined factors that could explain the relationship between socioeconomic factors and progress of MCC.

To enhance the process of comparing the findings between future studies assessing the socioeconomic inequalities in the progress of multiple chronic conditions, standardization of covariates should be considered. For example, education is probably a better indicator of socioeconomic status than income and occupation [36] as it is comparable between different countries and it also reflects early life socioeconomic status. These findings provide strong evidence that confirms the presence of socioeconomic inequalities in the incidence of multiple chronic conditions.

On the other hand, behavioural factors that were associated with the progress of MCC included diet, smoking, alcohol, physical activity [17,29] and BMI [16,17,27]. Studies also found that certain pre-existing chronic conditions such as diabetes, high blood pressure, coronary heart diseases, stroke and cancer contributed to progress of MCC [16,27,29,30].

To the best of our knowledge, this is the first systematic review that examined socioeconomic inequalities in the progress of multiple chronic conditions using population-based longitudinal studies. The review included different studies from different countries and database, confirmed that socioeconomic inequalities are persistent in the progress of MCC. The review also identified important gaps in understanding how socioeconomic factors relate to the progress of MCC which should inform the design of future research. Most of the included papers were rated as good quality with five of them including objective assessment of the outcomes. Finally, we highlighted some of the potential behavioural risk factors that contribute to the progress of MCC.

There are some limitations of this systematic review which should be noted. First, variations in socioeconomic factors, outcomes, covariates, and follow-up time impeded the pooling of the results, thus, it was not possible to conduct a meta-analysis. Second, although we searched the grey literatures, we could not identify any unpublished research. Like with any systematic review, there is always the risk of publication bias as studies with negative results are usually not published. Third, although all included studies reported the association between socioeconomic factors and progress of multiple chronic conditions, the focus of some of them was on other risk factors. Finally, some indicators of socioeconomic position such as homelessness were not used in any of the included studies. However, this is inevitable given the feasibility of repeated assessments over time of homeless population.

There are some implications of this review. The findings of this systematic review highlight the need for exploring social interventions to enhance the long-term prognosis of individuals with MCCs. Future research on inequality in the progress of MCC should explore explanatory pathways to demonstrate how socioeconomic factors influence chronic conditions over time and should also assess the impact of socioeconomic trajectories on MCC.

## Conclusion

Socioeconomic factors are longitudinally associated with the progress of multiple chronic conditions. In most of the studies, socioeconomic inequalities persisted even after accounting for behavioural risk factors and lifestyle. There is a need for further research exploring the different mechanisms for inequalities in the progress of multiple chronic conditions.

## Supporting information

S1 Checklist PRISMA 2020 checklist. (DOCX)
